# Trophoblastic debris modifies endothelial cell transcriptome *in vitro*: a mechanism by which fetal cells might control maternal responses to pregnancy

**DOI:** 10.1038/srep30632

**Published:** 2016-07-29

**Authors:** J. Wei, S. Y. Lau, C. Blenkiron, Q. Chen, J. L. James, T. Kleffmann, M. Wise, P. R. Stone, L. W. Chamley

**Affiliations:** 1 Department of Obstetrics and Gynaecology, The University of Auckland, New Zealand; 2 Department of Surgery, The University of Auckland, New Zealand; 3 Department of Molecular Medicine and Pathology, The University of Auckland, New Zealand; 4 Department of Biochemistry, University of Otago, New Zealand

## Abstract

The mechanisms by which the fetus induces maternal physiological adaptations to pregnancy are unclear. Cellular debris, shed from the placental syncytiotrophoblast into the maternal blood and phagocytosed by maternal endothelial and immune cells, may be one of these mechanisms. Here we show that trophoblastic debris from normal first trimester placentae induces changes in the transcriptome and proteome of endothelial cells *in vitro*, which might contribute to the adaptation of the maternal cardiovascular system to pregnancy. Trophoblastic debris also induced endothelial cells to transcribe placenta-specific genes, including the vasodilator hormone *CSH1*, thereby expanding the effective functional size of the placenta. Our data suggest that the deportation of trophoblastic debris is an important part of the complex network of feto-maternal communication.

A single multinucleated fetal cell, the syncytiotrophoblast, covers the human placenta. It is bathed in maternal blood and has a surface area of 11–13 m^2^. Like many cell types, the syncytiotrophoblast produces a range of vesicles but unique to the syncytiotrophoblast are syncytial nuclear aggregates (SNAs). It is on average approximately 70 μm in diameter and contains up to several hundred nuclei^1^. Collectively SNAs and other cellular material shed from the syncytiotrophoblast (diameter larger than 1 μm) are referred to as trophoblastic debris (TD) here^2^. Throughout pregnancy, vast numbers of membrane enclosed extracellular vesicles derived from the syncytiotrophoblast are extruded into the maternal circulation. Previously thought of simply as cellular junk, it is becoming increasingly clear that trophoblastic debris plays an important role in feto-maternal communication^3^.

Trophoblastic debris is rapidly cleared from the maternal circulation and vascular endothelial cells that line the blood vessels play a major role in this clearance^4^. Given there is a large amount of trophoblastic debris in contact with the maternal endothelium in all pregnancies^5^,^6^, it seems likely that this debris could alter endothelial cell function.

Here we investigated the transcriptional and proteomic changes that occur in human vascular endothelial cell line (HMEC-1) following exposure to trophoblastic debris from normal first trimester placentae and showed that trophoblastic debris alters the phenotype of endothelial cells in a manner that may help induce the cardiovascular changes necessary for normal pregnancy.

## Results

### Transcriptomic changes in HMEC-1 cells following exposure to trophoblastic debris for 2 or 21 hours

HMEC-1 cells were exposed to trophoblastic debris (TD) isolated from normal first trimester placentae for 2 or 21 hours. Differential expression analysis was carried out to compare the transcriptomic profile of HMEC-1 cells treated with TD to control HMEC-1 cells. TD up-regulated the expression of 119 genes and down-regulated the expression of 368 genes in HMEC-1 cells after 2 hours exposure compared to control HMEC-1 (Supplementary Table 1). Similarly, TD up-regulated the expression of 74 genes and down-regulated the expression of 187 genes in HMEC-1 cells compared to control after 21 hours exposure (Supplementary Table 2).

After 2 hours exposure to TD, the top 10 up-regulated genes in HMEC-1 cells were cytokine-related genes *CCL4*, *CCL3*, *CXCL3*; anti-apoptosis related genes *BIRC3* (Baculoviral IAP Repeat Containing 3); *TNFAIP3* (Tumor Necrosis Factor, Alpha-Induced Protein 3) and inflammatory response related genes *PTGS2*; *SELE*. The remaining 3 regulated genes were *MAP3K8; TIFA*; and *BCL6B*. However, none of these 10 genes were in the top 10 up-regulated genes of TD exposed HMEC-1 cells for 21 hours.

After 21 hours exposure to TD, the top 10 up regulated genes encoded the hormones: *CSH1///CSH2* (chorionic somatomammotropin hormone 1&2; also known as human placental lactogen, hPL); *CGA* (alpha chain of chorionic gonadotropin, also known as αHCG) and *IGF2///INS-IGF2* (Insulin-like growth factor 2); secreted extracellular signals: *ANGPTL4*; *SPP1*; *TFP12* and *MMP1*; the metabolism related genes: *PLIN2* and *PDK4*.

**Figure 1:**
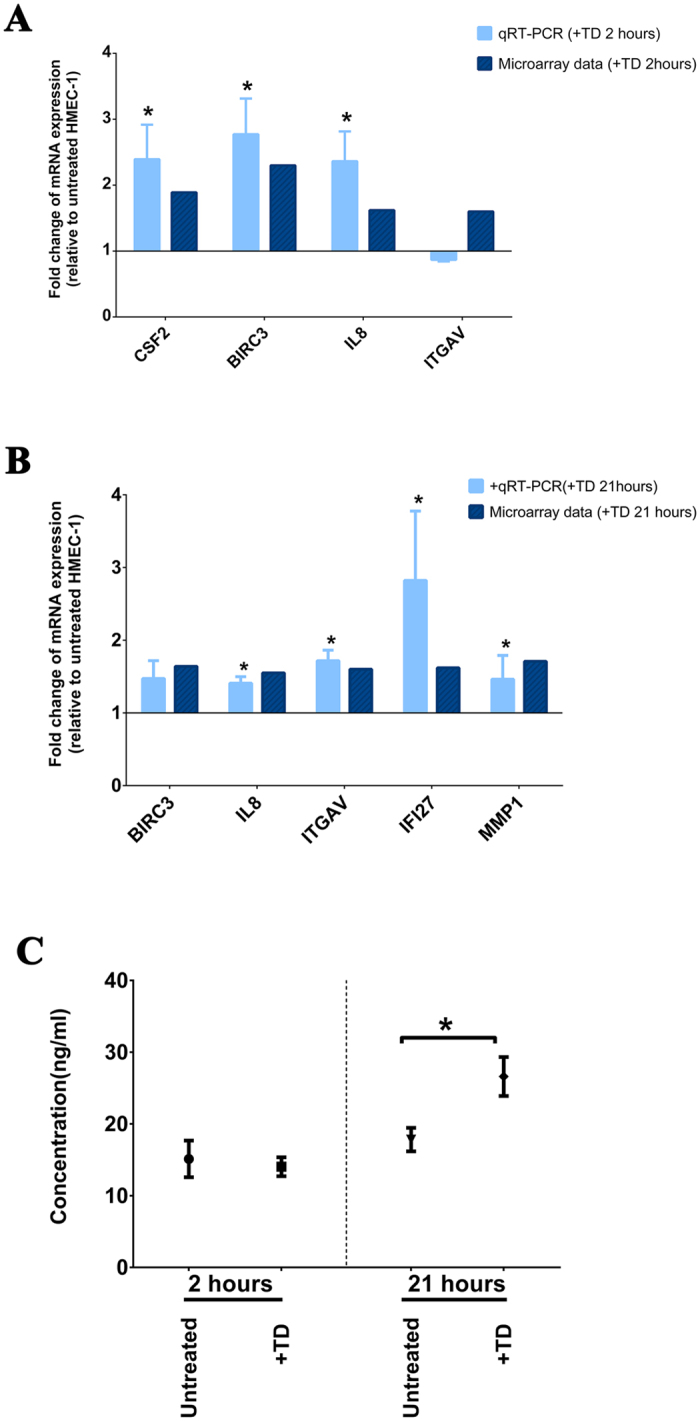
Quantitative RT-PCR Validation of microarray results. (**A**) The expression of four genes (*CSF2*, *BIRC3*, *IL8*, and *ITGAV*) in HMEC-1 cells by trophoblastic debris (+TD) following 2 hours exposure was validated by qRT-PCR. The expression was normalized to the geometric mean of housekeeping genes: *RPLP0*; *ACTB* and *PPIA*. Bar graphs represent the fold change of the mRNA expression relative to the control HMEC-1 cells by either qRT-PCR or microarray. Data are presented as mean ± SEM. *Indicates p-value < 0.01. (**B**) The expression of five genes (*BIRC3*, *IL8*, *ITGAV*, *IFI27* and *MMP1*) in HMEC-1 cells by trophoblastic debris (+TD) following 21 hours exposure was validated by qRT-PCR. The expression was normalized to the geometric mean of housekeeping genes: *RPLP0*; *ACTB* and *PPIA*. Bar graphs represent the fold change of the mRNA expression relative to the control HMEC-1 cells by either qRT-PCR or microarray. Data are presented as mean ± SEM. *Indicates p-value < 0.01. (**C**) Levels of IL-8 in conditioned media from HMEC-1 cells after 2 and 21 hours exposure to trophoblastic debris (TD) (collected from 6 individual placentae) were quantified by ELISA. Data are presented as mean ± SEM. *p < 0.05.

In order to validate the microarray data, we examined the expression of six genes (*CSF2*, *BIRC3*, *IL8*, *ITGAV*, *IFI27* and *MMP1*) by quantitative RT-PCR (qRT-PCR). The expression of *CSF2*, *BIRC3* and *IL8* was confirmed to be significantly up-regulated in HMEC-1 cells by TD (+TD) after 2 hours of exposure (p-value < 0.01, Fig. 1A). The expression of *IL8*, *IGTAV*, *IFI27* and *MMP1* was also validated to be significantly elevated in HMEC-1 cells exposed to TD for 21 hours compared to control HMEC-1 cells (p-value < 0.01, Fig. 1B). The up-regulation index determined by qRT-PCR was similar to the fold change shown in the microarrays, both of which were moderate.

Since both microarray and qRT-PCR analysis suggested that the expression of *IL8* mRNA was significantly increased in HMEC-1 cells exposed to TD, we examined the amount of IL-8 in the conditioned media from HMEC-1 cells that had been exposed to TD for either 2 or 21 hours. The ELISA analysis confirmed that levels of secreted IL-8 from HMEC-1 cells exposed to TD for 21 hours were significantly increased (26.61 ± 2.71 ng/mL) when compared to untreated HMEC-1 control at 21 hours (17.81 ± 1.64 ng/mL, p-value < 0.05) (Fig. 1C).

### Functional Annotation Clustering analysis

The enriched functional pathways based on the differentially regulated gene lists of HMEC-1 cells exposed to trophoblastic debris (TD) for 2 or 21 hours are shown in Table 1. At 2 hours, the most enriched pathway was cytokines, with 15 genes up regulated including many chemokines while leukocyte migration was also a highly enriched pathway. However, these pathways were no longer enriched at 21 hours. Rather, genes associated with blood vessel development were enriched at this later time point. Furthermore, genes encoding hormones were up regulated after 21 hours exposure to TD, including genes encoding hormones that would traditionally be considered to be placenta-specific such as *CSH1* and *CSH2*, *PSG3* (Pregnancy specific beta-1-glycoprotein 3), *PSG9* (Pregnancy specific beta-1-glycoprotein 9) as well as *CGA*.

### Proteomic changes in HMEC-1 cells exposed to trophoblastic debris for 24 hours

Comparison of the proteomes of untreated HMEC-1 cells to the HMEC-1 cells exposed to trophoblastic debris (TD) using isobaric tags for relative and absolute quantitation (iTRAQ) analysis identified a total of 3612 proteins in both groups with false discovery rate less than 1%. The average coefficient of variation (CV) of the measured fold change of relative protein abundances between three replicates was 20.3 +/− 17.5%. Using PANTHER classification system, 1606 genes were identified from the protein GI accession codes. A total of 59 proteins were significantly altered (by a fold change of at least 1.2) in HMEC-1 cells exposed to TD compared to controls, with 49 proteins up-regulated and 10 proteins down-regulated (Table 2, Supplementary Table 3). Among these 59 proteins, 5 “placenta-specific” proteins (CSH1///CSH2; PSG1; PSG3; PSG4 and STS (steroid sulfatase) were found to be up regulated in HMEC-1 cells after exposure to TD. When the results of transcriptomic and proteomic analyses were compared, the products of eight genes were confirmed to be up-regulated at both the mRNA and protein levels in HMEC-1 cells by TD. These proteins were: ITGAV (integrin, alpha V); ICAM1 (intercellular adhesion molecule 1); CSH1///CSH2, ANGPTL4, and PLIN2 (perilipin 2), TFPI2, S100P and PSG3. Based on the functional annotation using the DAVID bioinformatics tool^7^, they were involved in cell migration (the controlled self-propelled movement, GO:0016477) - ITGAV, ICAM1 and S100P; extracellular region (gene products secreted from a cell and released into the interstitial fluid or blood, GO:0005576) - PLIN2, TFPI2, CSH1, PSG3, ANGPTL4 and ICAM-1 and female pregnancy (The set of physiological processes that allow an embryo or fetus to develop, GO:0007565) - CSH1///CSH2 and PSG3.

### The effect of trophoblastic debris on the HMEC-1 cells angiogenic capacity *in vitro*.

**Figure 2:**
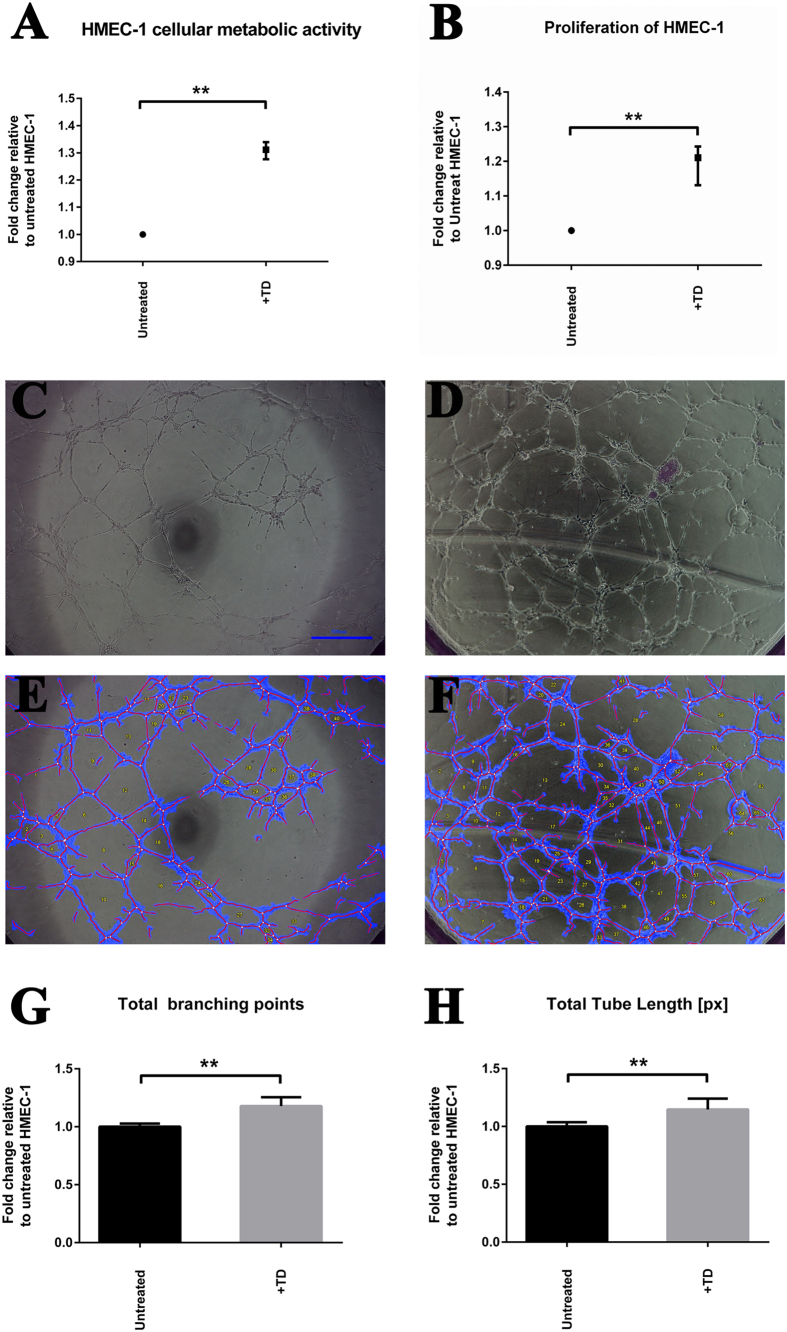
The proliferation and angiogenic capacity of HMEC-1 cells exposed to trophoblastic debris. (**A**) The cellular metabolic capacity of HMEC-1 cells treated with trophoblastic debris (TD, collected from 9 individual placentae) for 48 hours was investigated by AlamarBlue cell viability reagent. Data are presented as median ± interquartile range with p < 0.0001. (**B**) Cellular DNA contents of HMEC-1 cells treated with TD (isolated from 6 individual placentae) for 48 hours were investigated with CyQUANT cell proliferation assay. Data are presented as median ± interquartile range with p < 0.005. (**C**) Representative light microscope image of untreated HMEC-1 on growth factor reduced (GFR) matrigel at 4 hours; bar represents 500 μm. (**D**) Representative light microscope image of HMEC-1 treated with TD on GFR matrigel; bar represents 500 μm. (**E**) Representative automated images of untreated HMEC-1 using WimTube software. (**F**) Representative automated images of HMEC-1 treated with TD using WimTube software. (**G**) Total branching points generated from images of HMEC-1 cells and HMEC-1 cells treated with TD (n = 3 independent experiments). Data are presented as mean ± SD. p < 0.005. (**H**) Total tube length generated from images of HMEC-1 cells and HMEC-1 cells treated with TD (n = 3 independent experiments). Data are presented as mean ± SD. p < 0.005.

The functional annotation clustering analysis indicated that the effect of trophoblastic debris (TD) induced genes involve in the biological pathway of “blood vessel development” in HMEC-1 cells (Table 1). We further examined this possibility by measuring the effect of TD on HMEC-1 cell proliferation and tube formation ability *in vitro*. The cellular metabolic ability of HMEC-1 cells treated with TD was significantly increased by 31.1% ± 1.1% (p < 0.001, n = 9) compared to untreated HMEC-1 cells (Alamar Blue, Fig. 2A). The cellular DNA content of HMEC-1 cells treated with TD was significantly increased by 19.3 ± 2.4% (p < 0.03, n = 6) compared to untreated HMEC-1 cells (CyQuant cell proliferation assay, Fig. 2B). Exposure to TD significantly increased the number of HMEC-1 tubule branching points compared to control HMEC-1 (191.8 ± 11.8 vs. 161 ± 8, p < 0.02, n = 8, Fig. 2C–G). HMEC-1 exposed to TD formed significantly longer total length of tubes as compared to untreated HMEC-1 (20,669 ± 1,287 vs. 17,838 ± 1,328 pixels, p < 0.02, n = 8, Fig. 2C–F,2H).

### Confirmation that trophoblastic debris induced *de novo* expression of “placenta-specific” gene expression by HMEC-1 cells

**Figure 3:**
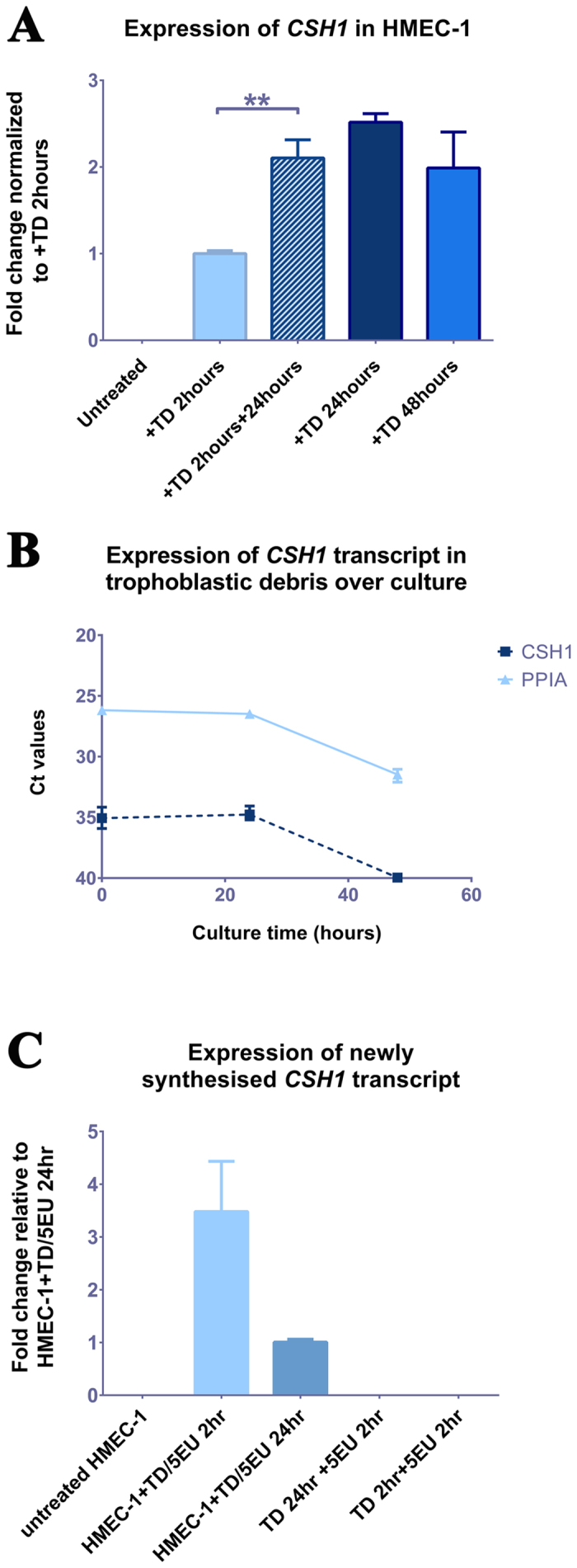
*De novo* expression of *CSH1* mRNA by HMEC-1 cells exposed to trophoblastic debris. (**A**) Expression of *CSH1* mRNA in HMEC-1 cells exposed to trophoblastic debris (+TD, isolated from 3 individual placentae) for 0, 2, 24 or 48 hours and kept in culture for another 24 hours after withdrawal of a 2 hour exposure. The expression was normalized to the geometric mean of housekeeping genes: *RPLP0*; *ACTB* and *PPIA*. Data are presented as Mean ± SD, P < 0.005. (**B**) Ct values of *CSH1* and housekeeping gene *PPIA* in trophoblastic debris (isolated from 3 individual placentae) continuously incubated alone for 0, 21 and 48 hours. Note a higher Ct value = low RNA abundance. (**C**) Newly synthesised RNA extracted from HMEC-1 co-cultured with trophoblastic debris (TD, isolated from 2 individual placentae) for 2 or 24 hours and the matched trophoblastic debris cultured alone for 2 or 24 hours. Expression of *CSH1* mRNA was normalized to housekeeping genes (*PPIA* and *ACTB*). Data are presented as Mean ± SD.

Both the microarray and iTRAQ analyses suggested that following exposure to trophoblastic debris (TD), HMEC-1 cells expressed transcripts considered to be “placenta-specific” at both gene and protein level. Therefore we conducted a closer examination of the expression, by HMEC-1 cells, of the placenta specific gene product *CSH1*. Quantification of the mRNA for *CSH1* indicated that HMEC-1 cells cultured with TD continuously for 2, 24 and 48 hours, increased their expression of *CSH1* mRNA over the 48 hour time course (Fig. 3A). In contrast, the level of *CSH1* transcripts in TD (from the same placentae) incubated without HMEC-1 cells declined to undetectable levels by 48 hours (Fig. 3B).

To further investigate whether the mRNA for *CSH1* was originating from TD or if expression was induced in the HMEC-1 cells, HMEC-1 cells were exposed to TD for 2 hours then washed the debris off and continued the culture for a further 24 hours. These HMEC-1 cells increased their expression of *CSH1* despite removing TD (Fig. 3A).

To finally confirm whether this *CSH1* transcript was freshly synthesised in HMEC-1 cells or delivered directly from the TD, newly synthesised RNA from either HMEC-1 cells that been exposed to TD or TD alone was isolated using the Click-iT^®^ Nascent RNA Capture Kit. Quantitative RT-PCR analysis confirmed HMEC-1 cells exposed to TD for either 2 or 24 hours synthesised nascent *CSH1* transcript. In contrast, TD alone in the culture did not transcribe new *CSH1* mRNA (Fig. 3C).

## Discussion

Trophoblastic debris is rapidly cleared from the maternal vasculature by an unclear mechanism, but it is likely that this mechanism involves maternal immune and endothelial cells^1^,^8^. We have previously studied changes of individual proteins in endothelial cells (and immune cells) in response to trophoblastic debris^9^,^10^ and were interested to understand the broader nature of the response of endothelial cells to trophoblastic debris.

Endothelial cells presented a moderate but dynamic response to trophoblastic debris with the expression of over 700 significantly regulated genes across both time points, but with only 17 genes with overlapping changes at both 2 and 21 hours. That some genes were regulated at 2 hours but not at the later time point is consistent with the known dynamic response of endothelial cells to various activators^11^. Although expression of many genes was mildly regulated in the microarray experiments, this regulation was consistent as confirmed by qRT-PCR and also at the protein level as shown by the changes in IL-8. While the level of *IL8* transcripts was significantly increased at both 2 and 21 hours after exposing the endothelial cells to trophoblastic debris, the protein levels were only significantly increased 21 hours after exposure. This is likely to represent the time delay required for the endothelial cells to synthesize and secrete adequate amounts of IL-8 for detection in the ELISA.

To complement our transcriptomic study we also conducted an unbiased analysis of global changes in the proteome of endothelial cells exposed to trophoblastic debris (using iTRAQ) and found significant changes in 59 proteins. While there was far from complete overlap in the results from the transcriptomic and proteomic studies, this is to be expected given the widely differing sensitivities of the two techniques employed and it was very encouraging to find that there several there were parallel changes in the transcripts and protein products of several genes.

The mechanisms used by the feto-placental unit to induce adaptation of the maternal cardiovascular system to pregnancy are not entirely understood. Here we have shown that trophoblastic debris has the ability to alter the behaviour of vascular endothelial cells promoting both their proliferation and branching of tubules *in vitro*. Interestingly, the mRNA expression for several angiogenic chemokines (*CXCL1*, *CCL3*, *CCL2*, *IL8*, *CXCL3*, *CXCL2*, *CCL4*, *IL1A*) and a few hormones^12^,^13^ involved in angiogenesis (*CSH1*, *LEP*, *CGA*, *CSH2*, *INS-IGF2*, *IGF2*, *GAL*) were up-regulated in endothelial cells following exposure to trophoblastic debris, of which IL-8 has been shown to have direct effect on endothelial cells proliferation and can regulate angiogenesis^14^. Further, the prominent up-regulation of the negative regulators of apoptosis including, *BIRC3* and *TNFAIP3* (Table 1) as part of the endothelial cell response to trophoblastic debris is also likely to promote endothelial cell survival.

Others have previously suggested that microparticles derived from syncytiotrophoblast (STBM) inhibit endothelial cell proliferation and induce apoptosis^15^,^16^,^17^. However, the microvesicles were obtained by mechanical disruption of term placentae, a technique which has been shown to produce microparticles with non-physiological actions and furthermore since the cardiovascular adaptations to pregnancy occur in the first trimester the use of term placentae-derived TD or microparticles would be inappropriate to address the issue of maternal cardiovascular adaptation^18^. In support of our findings, a recent study demonstrated that placental exosomes, a smaller 100 nM sized vesicle population; from first trimester healthy placentae had the most obvious effect in promoting endothelial cell migration^3^. While we haven’t identified the mechanism, our results suggest that trophoblastic debris might contribute to vasculature development and regeneration of the endothelium in response to the rapid increases in blood volume and vasodilation^19^,^20^.

During pregnancy it is reported that there are increased levels of pro-inflammatory markers compared to the non-pregnant state and that there is a further increase in the inflammatory markers in preeclampsia^21^,^22^,^23^. Generally it is considered that these markers are produced by the maternal immune cells, but our data show that maternal endothelium may also contribute to this inflammatory response during pregnancy. This supports the previous reports by others showing that that microparticles from lymphocytes or platelets can induce production of inflammatory and pro-coagulant mediators by endothelial cells^24^. Here we show that trophoblastic debris (which is generally larger in size than microparticles) induces the production of inflammatory cytokines and chemokines by endothelial cells in both transcriptional and protein level.

We were intrigued by the observation that exposing endothelial cells to trophoblastic debris resulted in the endothelial cells apparently expressing placenta-specific genes (e.g. *CSH1/CSH2*, *CGA*, *PSG3*, and *PSG9*). This result suggests that trophoblastic debris might be a mechanism that the fetus uses to expand the effective production capacity for placental hormones/products without the need to expend energy on expanding the size of the placenta itself. Therefore we confirmed up-regulation of these genes at the protein level using an unbiased proteomics approach, but the possibility remained that this apparent expression of placenta-specific genes by the endothelial cells at both the RNA and protein level was due to contamination by tightly adherent trophoblastic debris that was not removed by the washing steps. However, that possibility seems very unlikely for several reasons: 1) the RNA in trophoblastic debris is mostly RNA fragments and is of low concentration (data not shown); 2) after two hours of exposure to trophoblastic debris and subsequent removal of the debris, endothelial cells still continued to increase the expression of *CSH1 *mRNA for another 24 hours; 3) endothelial cells exposed to trophoblastic debris steadily expressed *CSH1* mRNA for up to 48 hours while debris “cultured” for 48 hours in the absence of endothelial cells failed to express *CSH1* mRNA. We further confirmed that the trophoblastic debris-exposed endothelial cells expressed nascent *CSH1* by isolating the fraction of newly synthesised RNA from the endothelial cells after exposure to trophoblastic debris. In contrast, trophoblastic debris “cultured” without endothelial cells did not produce new *CSH1* mRNA. In combination these results suggest that regulatory factors, either RNAs or proteins, were transferred to the endothelial cells from the trophoblastic debris and induced the transcription of placental genes in the endothelial cells. Of biologic relevance to our study, it has been shown that CSH1 can induce endothelium dependent vasodilation through nitric oxide production^25^ which, since nitric oxide is key physiologic vasodilator, may be important in the normal physiologic adaptation of the maternal cardiovascular system to pregnancy.

Others have recently shown that trophoblastic exosomes can transfer viral resistance via microRNAs from the placenta to other cell types (including endothelial cells and fibroblasts)^26^ and this could be one mechanism whereby trophoblastic debris induced phenotypic changes in endothelial cells in this work. Regardless of the mechanism by which trophoblastic debris induced *de novo* placental gene expression in endothelial cells, this finding indicates that trophoblastic debris could be a mechanism used by the feto-placental unit to influence maternal systems.

In this work we have examined the effects of trophoblastic macro-debris on endothelial cells. In recent years most researchers working in this field have focused their attention on the role of trophoblastic micro- and nano-vesicles/particles which are also part of the spectrum of placental extracellular vesicles^3^,^16^,^18^,^27^,^28^,^29^,^30^. That several of our findings on the effects of trophoblastic macro-debris on endothelial cells parallel the published effects of trophoblastic micro- and nano-vesicles/particles suggests that regardless of the size of the debris, and therefore potentially the mechanism by which it is produced, trophoblastic debris from normal placentae may have the ability to modify both transcriptome and the function of the maternal endothelium^3^. These effects may co-ordinate maternal vascular endothelial cells to react quickly to the stress caused by maternal blood volume expansion and increased shear stress during the maternal vascular adaptation to pregnancy.

**Figure 4:**
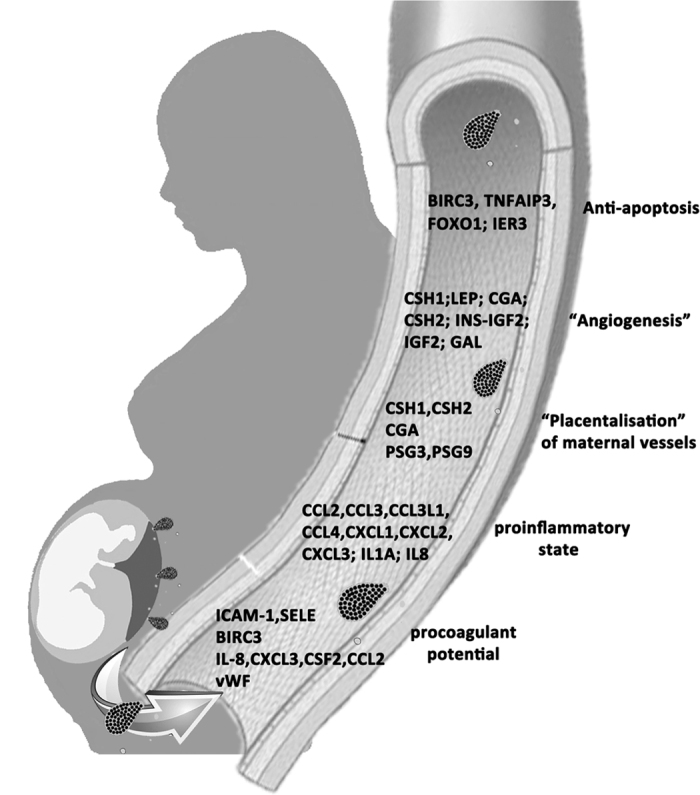
The effect of trophoblastic debris on maternal endothelial cells. Trophoblastic debris enters maternal circulation and might contribute to maternal adaptations through the effect on endothelial cells, including anti-apoptosis, “angiogenesis”, “placentalisation” of maternal vessels, proinflammatory effect.

In conclusion, while for many years it was thought that trophoblastic debris was simply part of a waste disposal system and that the debris was of little value, it seems highly likely that trophoblastic debris is a substantial contributor to the feto-maternal communication that is essential to the normal maternal cardiovascular adaptation to pregnancy (Fig. 4).

## Methods

### Collection of placentae

This work received approval from the Auckland Regional Health and Disabilities Ethics Committee (NTX/12/06/057/AM03). All procedures were performed following relevant guidelines and in accordance with the appropriate regulations. All patients were informed and signed the content forms before donating the human tissue. First trimester placentae (n = 38) ranging from 8 to 12 weeks of gestation were collected following elective surgical termination of pregnancies.

### Cell culture

Human endothelial cells from the Human Microvascular Endothelial Cell line (HMEC-1) were cultured in MCDB 131 medium supplemented with 10% FBS, 1% L-glutamine and 1% Penicillin/Streptomycin (Life technologies, Thermo Fisher Scientific). All HMEC-1 cells were used at passages lower than 15.

### Preparation of trophoblastic debris

Trophoblastic debris from cultured placental explants was isolated as previously described^31^. Explants of approximately 400 mg wet weight were cultured in Netwell inserts (Corning 3478) overnight. Media from beneath the chamber was centrifuged at 2000 × g for 5 minutes to isolate the trophoblastic debris. Contamination red blood cells and leukocytes were depleted using red blood cells lysis buffer and CD45 Dynabeads (Invitrogen, Life Technologies). The protein content of trophoblastic debris was determined with a Pierce BCA Protein Assay Kit (Thermo Fisher Scientific)^31^.

### RNA extraction

HMEC-1 cells were grown until 80% confluence and then treated with 60 μg total trophoblastic debris protein per mL (isolated from 8 individual placentae) for the indicated length of time. After washing twice with PBS, the cells were lysed with 1 mL TRIzol reagent (Invitrogen, Life Technologies). Total RNA was purified by PureLink^®^ RNA Mini Kit (Invitrogen, Life Technologies). The quantity and quality of total RNA was measured by NanoDrop ND-1000 Spectrophotometer (Thermo Fisher Scientific) and Experion automated electrophoresis system using Experion RNA StdSens Analysis Kit (BioRad, Hercules, CA, USA), respectively.

### Newly synthesised RNA isolation

The newly transcribed RNA was isolated using Click-iT^®^ Nascent RNA Capture Kit (Life Technologies, C-10365). Briefly, HMEC-1 cells co-cultured with 60 μg total trophoblastic debris protein per mL (isolated from 2 individual placentae) or matched trophoblastic debris alone was incubated in the presence of 0.2 mM 5-ethynyl Uridine (5EU) at 37 °C, 5% CO_2_ for either 2 or 24 hours. Total RNA was extracted using TRIzol and purified by PureLink^®^ RNA Mini Kit as mentioned before. 5EU labeled RNA was then biotinylated by Biotin Azide at room temperature for 30 minutes before precipitated at −70 °C overnight. Biotinylated RNA was bound to streptavidin conjugated magnetic beads and isolated by DynaMag™-2 Magnet. Isolated RNA was used in consequent cDNA synthesis and qRT-PCR.

### Microarray analysis

Array profiling was performed using Affymetrix PrimeView Human Gene Expression 3′IVT arrays (Affymetrix, CA, USA). Biotin-labelled aRNA was prepared using the GeneChip 3′IVT Express kit (Affymetrix) using manufacturer’s protocols. Labelled RNA was hybridised onto GeneChip microarrays as per manufacturer’s protocols prior to scanning. This was performed as a service by New Zealand Genomics Ltd. After scanning, CEL files were imported into the statistical software program R. The data was pre-processed and normalized using RMA from the R package ‘affy’ in Bioconductor^32^,^33^. The package Limma was used to check for differential expression, and an empirical Bayes method used to moderate the t-statistic. The Benjamini-Hochberg method was used to adjust the p-values for multiple sampling. The cut-off values used in this study are log2 fold change = 0.5; p-value < 0.05.

DAVID Bioinformatics Resources 6.7 was used to perform functional annotation clustering for those genes that were significantly regulated in endothelial cells following exposure to trophoblastic debris^34^,^35^.

### qRT-PCR analysis

1 μg RNA from each sample was reverse transcribed into cDNA by SuperScript^®^ III First-Strand Synthesis SuperMix (Invitrogen, Life Technologies) following the manufacturer’s instructions. qRT- PCR was performed using Platinum^®^ SYBR^®^ Green qPCR SuperMix-UDG w/ROX (Invitrogen, Life Technologies). Relative expression of *CSF2*, *BIRC3*, *IL8*, *ITGAV*, *IFI27*, *MMP1* and *CSH1* mRNAs were normalized to four housekeeping genes: *UBC*, *RPLP0*, *ACTB* and *PPIA*. The sequences of the qRT-PCR primer pairs for each gene are shown in Supplementary Table 4.

### Protein Extraction, Digestion, iTRAQ Labeling and LC-MS/MS

HMEC-1 were grown in six well plates until 80% confluence and then treated with 60 μg total trophoblastic debris protein per mL isolated from 3 individual placentae for 24 hours. After washings with PBS, 250 μl of ice-cold iTRAQ compatible buffer (0.5 M triethylammonium bicarbonate (TEAB) buffer +0.01% sodium dodecyl sulphate) was added to each well and incubated on ice for 30 minutes with intermittent vigorous pipetting. The lysate was sonicated in an ice-cold water bath at 40% amplitude for 5 periods of 30 seconds, with 30 seconds of rest in between each sonication period, followed by centrifugation at 17 000 × g for 10 minutes. 10 μl of nuclease (Novagen^®^, Merck Millipore) was added to the supernatant and incubated on ice for 30 minutes before storing the samples at −80 °C.

For each sample, a total of 100 μg of protein were purified and buffer exchanged essentially following the filter-aided sample preparation (FASP) protocol^36^ using centrifugal filter units with a 3 kDa cut-off membrane (Amicon Ultra 0.5 mL; Merck Millipore, Darmstadt, Germany). In brief SDS was removed by three consecutive urea washes followed by two consecutive washes in 100 mM TEAB. Cysteine thiols were reduced on filter with 5 mM tris (2-carboxyethyl)phosphine in 100 mM TEAB at 50 °C for 15 min., washed once with 100 mM TEAB and then blocked with 200 mM methyl methanethiosulfonate in the dark for 30 min. The reducing and blocking reagents were removed by two washes in 100 mM TEAB and trypsinised with 4 μg of trypsin (sequencing grade trypsin; Promega, Madison, WI) in a total volume of 50 μl of 100 mM TEAB per sample. Proteins were digested on filter at 37 °C overnight. Tryptic peptides were then iTRAQ labelled according to the manufacturer’s instructions (AB Sciex Inc., Foster City). The control samples were labelled with iTRAQ tags 117, and the corresponding trophoblastic debris exposed samples were labelled with iTRAQ tags 114. After pooling of labelled samples, peptides were dried down to 50 μL using a centrifugal vacuum concentrator, reconstituted in 1 ml of 0.2% formic acid in water and purified by Sep-Pak (Waters) C-18 solid phase extraction. Purified peptides were dried using a centrifugal vacuum concentrator.

The pooled iTRAQ labelled sample were fractionated into 24 fractions by off-gel isoelectric focusing along a pH gradient from 4–10 using an Agilent OFFGEL Frationator (Agilent Technologies) according to the manufacturer’s instructions. Each of the 24 fractions was concentrated using a centrifugal vacuum concentrator and reconstituted in 5% (vol/vol) acetonitrile (ACN), 0.2% (vol/vol) formic acid in water to a total volume of 50 μL. Each fraction was analysed in duplicates by nanoflow liquid chromatography-coupled tandem mass spectrometry (LC-MS/MS) using a Ultimate 3000 nanoflow uHPLC system (Dionex, Thermo Scientific, San Jose, CA) inline coupled to the nanospray source of a LTQ Orbitrap XL mass spectropmeter (Thermo Scientific, San Jose, CA). Therefore 20 μl of each fraction were loaded onto an in-house packed trap column (100 μm inner diameter fused silica tubing packed with 5 μm C18 beads on a length of 2 cm) and separated on an in-house packed emitter tip column (75 um ID PicoTip fused silica tubing (New Objectives, USA) packed with 3 μm C-18 beads on a length of 12 cm). Two different LC-gradients of solvent A (0.2% (vol/vol) formic acid inwater) in solvent B (0.2% (vol/vol) formic acid in acetonitrile) were used for each of the two replicate analyses to increase peptide identification and the number of quantification counts per protein. Gradient 1 was developed from i) 5 to 12% B over 4 min. ii) 12 to 30% B over 70 min. iii) 30 to 45% B over 8 min. and iv) 45 to 99% B over 4 min. followed by column washing and re-equilibration. For the second analysis step 2 from 12 to 30% B was shortened to 30 min.; all other steps were kept constant between the replicate analyses.

The mass spectrometer was operated in data-dependent mode acquiring one full MS spectrum over a mass range of m/z 400–2000 in the Orbitrap analyser at a resolution of 60,000. The strongest 4 signals were selected for CID (collision induced dissociation)-MS/MS in the LTQ ion trap at a normalised collision energy of 35%. The same 4 precursors were selected again for HCD (high energy collision induced dissociation)-MS/MS at a relative collision energy of 52% and subsequent fragment ion measurement in the Orbitrap analyser at a resolution of 7500 at m/z 400. Dynamic exclusion was enabled with 2 repeat counts during 60 seconds and an exclusion period of 240 seconds.

### Proteomics Data Analysis

Raw data were processed through the Proteome Discoverer software (Thermo Scientific) using default settings for peak processing. For protein identification, both CID and HCD spectra were searched against the human reference amino acid sequence database using the Mascot (http://www.matrixscience.com) and SequestHT (Thermo Scientific, San Jose, CA) search engines. The search was set up for full tryptic peptides with a maximum of 3 missed cleavage sites. Methylthiocysteine, oxidised methionine and iTRAQ labelling on Lys, Tyr and the peptide N-terminus were included as variable modifications. The precursor mass tolerance threshold was 10 ppm and the maximum fragment mass error 0.8 Da. Only high confidence peptide hits at a false discovery rate of <1% was considered for protein identification and quantification. The score threshold for identification was adjusted to achieve an estimated FDR of <1% using the percolator algorithm^37^. We only accepted protein identifications with at least two significant peptide hits per protein group. Reporter ion quantification was performed on HCD spectra considering only unique peptides for each protein groups for quantification. The ratios were normalized on the median of 50 protein identifications.

Proteins altered in treated endothelial cells relative to controls <0.8 fold or >1.2 fold in at least two out of the three biological replicates were considered differentially altered. The data were characterized using the PANTHER classification system (www.patherdb.org).

### Quantification of IL-8 levels in the conditioned media from HMEC-1 cells exposed to trophoblastic debris.

HMEC-1 cells were exposed to paired trophoblastic debris (n = 6 individual placentae) for either 2 or 21 hours. The amount of IL-8 in the conditioned media was quantified using Human IL-8 ELISA Set (BD OptEIA™, 555244) following the manufacturer’s instructions. Duplicate wells of each sample were measured and the inter-assay variation was less than 10%.

### Endothelial cell proliferation

HMEC-1 cells were cultured in MCDB 131 medium supplemented with 10% FBS until 80% confluence and then treated with 60 μg total trophoblastic debris protein per mL (isolated from 15 individual placentae). After co-culture for 48 hours, the cellular metabolic capacity and cellular DNA contents of HMEC-1 cells were assessed by AlamarBlue^®^ Cell Viability Assay and CyQUANT^®^ Cell Proliferation Assays (Life Technologies) respectively. All experiments had three technical replicates.

### Tube formation assay

HMEC-1 were grown until 80% confluence and then treated with 60 μg total trophoblastic debris protein per mL (isolated from 3 separate placentae) for 24 hours. HMEC-1 were trypsinized and seeded in ibiTreat Angiogenesis Slide (ibidi, 81506, Germany) coated with growth factor reduced matrigel (Corning, 354230) at the number of 10^4^ cells per well and incubated at 37 °C, 5% CO_2_ for 4 hours. All experiments had three technical replicates. The whole area of the wells was captured by Nikon’s inverted microscope (Eclipse Ti-E). Total branching points and total tube length were analyzed by WimTube software (ibidi, Germany).

### Statistical analysis

The statistical significance of the results was assessed by a Mann-Whitney U-test or a Kruskal-Wallis test (ANOVA) using the GraphPad Prism 6 software, with p < 0.05 being considered significant.

## Additional Information

**How to cite this article**: Wei, J. *et al.* Trophoblastic debris modifies endothelial cell transcriptome *in vitro*: a mechanism by which fetal cells might control maternal responses to pregnancy. *Sci. Rep.* **6**, 30632; doi: 10.1038/srep30632 (2016).

## Supplementary Material

Supplementary Information

## Figures and Tables

**Table 1:** Functional analysis of modulated gene pathways in HMEC-1 treated with trophoblastic debris for 2 and 21 hours.

Biological process	Count*	%†	P Value	Genes	Fold Enrichment‡	FDR§
2 hours						
Cytokine	15	12.6	4.00E-12	*CXCL1, CSF2, IL6, CCL3, CCL2, IL-8, CXCL3, CXCL2, CCL4L1, CCL4L2, GREM1, CCL4, LIF, TNFSF10, CCL3L1, CCL3L3, IL1A*	13.74	5.02E-09
Anti- apoptosis	12	10.1	4.94E-05	*MEF2C, CSF2, IER3, IL6, CCL2, NUAK2, NFKBIA, FOXO1, PIM3, TNFAIP3, BIRC3, IL1A*	4.63	0.079
Regulation of transcription	36	30.3	9.04E-05	*MEF2C, ACVRL1, NFKBIA, FOXO1, SOX7, CITED4, CNOT4, LIF, REL, BHLHE40, SIK1, IRAK2, ICAM1, NFKBIZ, MAFF, ZNF563, IL6, ERG, EPAS1, CEBPD, ZNF440, RELB, KLF11, NDUFA13, NR4A1, NR4A3, TLE2, C14ORF43, ABCG1, PTHLH, EAF1, NAB2, IRF1, BCL6B, ZSCAN18, KLF4*	1.89	0.145
Leukocyte migration	6	5.0	5.59E-05		14.38	0.090
21 hours						
Extracellular region	22	28.9	7.94E-06	*CSH1, CGA, CSH2, IL-8, INS-IGF2, PSG3, IL32, IGF2, CTSS, GAL, MMP1, VCL, LEP, PSG9, PLIN2, SERPINE1, IL1B, LAMC2, SCG5, TFPI2, DST, ANGPTL4, SPP1*	2.74	0.009
Blood vessel development	6	7.9	74.87E-03	*IL-8, ITGAV, IL1B, MKL2, JUNB, ANGPTL4*	5.34	7.396
Response to external stimulus	6	7.9	7.37E-04	*LEP, IL-8, OSMR, INS-IGF2, SERPINE1, IGF2, SPP1*	8.23	1.152
Hormone activity	6	7.9	1.38E-04	*CSH1, LEP, CGA, CSH2, INS-IGF2, IGF2, GAL*	11.82	0.172

^*^Count: Number of genes in the regulated list that were involved in the indicated biological process.

^†^%: Percentage of the total genes in the annotation category.

^‡^Fold Enrichment: The ratio of the percentage of involved genes in total regulated genes compared to the background information. The high the score is, the more enrich the biological process is involved in treatment.

^§^FDR (False Discovery Rate): A statistic for multiple comparison correction.

**Table 2:** Identification of differentially expressed proteins in trophoblastic debris treated endothelial cells at 24 hours.

	Proteins
Up regulated	CKMT, HSD11B2, K2C8, S100P, CSH1/CSH2, TFPI2, HBG1, PSG1, PSG4, HBG2, KRT19, PSG3, STS, TF, CYB5R1, ANXA4, HBB, PAI2, S100A9, TFRC, KRT18, MAOA, ICAM1, PAI1, ANXA6, PSMD8, ATP6V0D1, SLC2A1, ITGB3, SBSPON, HBA, NFKB2, ECH1, ANGPTL4, VPP1, LNPEP, EDIL3, PLIN2, LAMP1, DYSF, AGRIN, SLC2A3, CYP, ITGAV, MPRIP, TGM2, MANF, NUDT5, NDUFAB1
Down regulated	IMMT, SLC25A3, VDAC2, RPL17, RPS13, FETUA, KRT10, KRT1, FAM162A, SCaMC-1
