# Supplementary material for: Trophoblastic debris modifies endothelial cell transcriptome in vitro: a mechanism by which fetal cells might control maternal responses to pregnancy
Source: Sci Rep. 2016 Jul 29;6:30632. doi: 10.1038/srep30632 (PMC4965770; doi:10.1038/srep30632)
Supplement: Supplementary Information [file srep30632-s1.doc]

**Trophoblastic debris modifies endothelial cell transcriptome *in vitro*: a mechanism by which fetal cells might control maternal responses to pregnancy**

Wei J^1*^, Lau S^1^, Blenkiron C^2,3^, Chen Q^1^, James JL^1^, Kleffmann T^4^, Wise M^1^, Stone PR^1^, Chamley LW^1^

1. Department of Obstetrics and Gynaecology, The University of Auckland, New Zealand
2. Department of Surgery, The University of Auckland, New Zealand
3. Department of Molecular Medicine and Pathology, The University of Auckland, New Zealand
4. Department of Biochemistry, University of Otago, New Zealand

*Corresponding author: [j.wei@auckland.ac.nz](mailto:j.wei@auckland.ac.nz)

**Supplementary table 1. Regulated genes of HMEC-1** cells after exposure to trophoblastic debris for 2 hours.

| **Probe Set ID** | **Gene Title** | **Gene Symbol** | **log2 fold change** | **p value** |
| --- | --- | --- | --- | --- |
| **11718982_s_at** | chemokine (C-C motif) ligand 4 /// chemokine (C-C motif) ligand 4-like 1 /// chemokine (C-C motif) ligand 4-like 2 | CCL4 /// CCL4L1 /// CCL4L2 | 2.824 | 0.000 |
| **11726316_at** | selectin E | SELE | 1.708 | 0.004 |
| **11718983_x_at** | chemokine (C-C motif) ligand 4 /// chemokine (C-C motif) ligand 4-like 1 /// chemokine (C-C motif) ligand 4-like 2 | CCL4 /// CCL4L1 /// CCL4L2 | 1.365 | 0.001 |
| **11720994_x_at** | chemokine (C-C motif) ligand 3 | CCL3 | 1.287 | 0.000 |
| **11722851_at** | baculoviral IAP repeat-containing 3 | BIRC3 | 1.217 | 0.001 |
| **11728476_a_at** | chemokine (C-X-C motif) ligand 3 | CXCL3 | 1.212 | 0.000 |
| **11724037_at** | prostaglandin-endoperoxide synthase 2 (prostaglandin G/H synthase and cyclooxygenase) | PTGS2 | 1.195 | 0.004 |
| **11722852_s_at** | baculoviral IAP repeat-containing 3 | BIRC3 | 1.157 | 0.001 |
| **11722850_a_at** | baculoviral IAP repeat-containing 3 | BIRC3 | 1.137 | 0.002 |
| **11736217_at** | mitogen-activated protein kinase kinase kinase 8 | MAP3K8 | 1.092 | 0.011 |
| **11720681_at** | TRAF-interacting protein with forkhead-associated domain | TIFA | 1.069 | 0.001 |
| **11718939_s_at** | tumor necrosis factor, alpha-induced protein 3 | TNFAIP3 | 1.056 | 0.002 |
| **11729456_a_at** | B-cell CLL/lymphoma 6, member B | BCL6B | 1.054 | 0.001 |
| **11720682_at** | TRAF-interacting protein with forkhead-associated domain | TIFA | 1.015 | 0.001 |
| **11758619_s_at** | leukemia inhibitory factor (cholinergic differentiation factor) | LIF | 0.989 | 0.000 |
| **11744128_x_at** | chemokine (C-X-C motif) ligand 2 | CXCL2 | 0.975 | 0.004 |
| **11716341_s_at** | ephrin-A1 | EFNA1 | 0.967 | 0.001 |
| **11744127_at** | chemokine (C-X-C motif) ligand 2 | CXCL2 | 0.966 | 0.003 |
| **11719366_s_at** | chemokine (C-X-C motif) ligand 1 (melanoma growth stimulating activity, alpha) | CXCL1 | 0.960 | 0.001 |
| **11754114_a_at** | chemokine (C-X-C motif) ligand 1 (melanoma growth stimulating activity, alpha) | CXCL1 | 0.954 | 0.001 |
| **11716071_s_at** | pim-3 oncogene | PIM3 | 0.938 | 0.000 |
| **11717994_a_at** | nuclear receptor subfamily 4, group A, member 1 | NR4A1 | 0.925 | 0.002 |
| **11728876_at** | colony stimulating factor 2 (granulocyte-macrophage) | CSF2 | 0.922 | 0.005 |
| **11763250_x_at** | chemokine (C-X-C motif) ligand 1 (melanoma growth stimulating activity, alpha) /// chemokine (C-X-C motif) ligand 2 | CXCL1 /// CXCL2 | 0.918 | 0.001 |
| **11745878_x_at** | nuclear factor of kappa light polypeptide gene enhancer in B-cells inhibitor, alpha | NFKBIA | 0.903 | 0.000 |
| **11719833_at** | myelin protein zero-like 2 | MPZL2 | 0.902 | 0.009 |
| **11746954_s_at** | chemokine (C-C motif) ligand 4 /// chemokine (C-C motif) ligand 4-like 1 /// chemokine (C-C motif) ligand 4-like 2 | CCL4 /// CCL4L1 /// CCL4L2 | 0.879 | 0.007 |
| **11718940_a_at** | tumor necrosis factor, alpha-induced protein 3 | TNFAIP3 | 0.875 | 0.003 |
| **11716384_at** | chemokine (C-C motif) ligand 2 | CCL2 | 0.874 | 0.000 |
| **11754106_a_at** | ephrin-A1 | EFNA1 | 0.856 | 0.002 |
| **11743136_x_at** | CCAAT/enhancer binding protein (C/EBP), delta | CEBPD | 0.850 | 0.003 |
| **11756746_s_at** | chromosome 11 open reading frame 17 /// NUAK family, SNF1-like kinase, 2 | C11orf17 /// NUAK2 | 0.812 | 0.000 |
| **11754035_a_at** | interferon regulatory factor 1 | IRF1 | 0.811 | 0.001 |
| **11744000_a_at** | nuclear factor of kappa light polypeptide gene enhancer in B-cells inhibitor, alpha | NFKBIA | 0.810 | 0.001 |
| **11728477_at** | chemokine (C-X-C motif) ligand 3 | CXCL3 | 0.794 | 0.012 |
| **11750174_a_at** | supervillin pseudogene | LOC645954 | 0.786 | 0.007 |
| **11755564_x_at** | chemokine (C-C motif) ligand 3-like 1 /// chemokine (C-C motif) ligand 3-like 3 | CCL3L1 /// CCL3L3 | 0.779 | 0.001 |
| **11715119_s_at** | C2 calcium-dependent domain containing 4B | C2CD4B | 0.778 | 0.007 |
| **11752869_s_at** | CCAAT/enhancer binding protein (C/EBP), delta | CEBPD | 0.777 | 0.008 |
| **11743135_s_at** | CCAAT/enhancer binding protein (C/EBP), delta | CEBPD | 0.764 | 0.013 |
| **11716733_at** | interferon regulatory factor 1 | IRF1 | 0.757 | 0.000 |
| **11744529_a_at** | pellino homolog 1 (Drosophila) | PELI1 | 0.756 | 0.001 |
| **11757894_x_at** | nuclear factor of kappa light polypeptide gene enhancer in B-cells inhibitor, alpha | NFKBIA | 0.717 | 0.003 |
| **11722141_at** | v-rel reticuloendotheliosis viral oncogene homolog B | RELB | 0.717 | 0.001 |
| **11727503_at** | SRY (sex determining region Y)-box 7 | SOX7 | 0.695 | 0.001 |
| **11726498_at** | leukemia inhibitory factor (cholinergic differentiation factor) | LIF | 0.690 | 0.001 |
| **11754026_a_at** | interleukin 8 | IL8 | 0.686 | 0.029 |
| **11718841_s_at** | interleukin 8 | IL8 | 0.685 | 0.002 |
| **11763115_a_at** | immunoglobulin heavy constant gamma 1 (G1m marker) /// immunoglobulin heavy constant mu /// ig heavy chain V-I region V35-like | IGHG1 /// IGHM /// LOC100133862 | 0.678 | 0.033 |
| **11743007_at** | nuclear factor of kappa light polypeptide gene enhancer in B-cells inhibitor, epsilon | NFKBIE | 0.677 | 0.000 |
| **11716299_a_at** | integrin, alpha V (vitronectin receptor, alpha polypeptide, antigen CD51) | ITGAV | 0.677 | 0.022 |
| **11723213_a_at** | myocyte enhancer factor 2C | MEF2C | 0.674 | 0.017 |
| **11747833_a_at** | Kruppel-like factor 11 | KLF11 | 0.674 | 0.004 |
| **11755981_a_at** | heparin-binding EGF-like growth factor | HBEGF | 0.673 | 0.010 |
| **11733240_at** | interleukin-1 receptor-associated kinase 2 | IRAK2 | 0.672 | 0.007 |
| **11739046_s_at** | multimerin 2 | MMRN2 | 0.670 | 0.012 |
| **11763226_x_at** | interleukin 8 | IL8 | 0.669 | 0.032 |
| **11722768_s_at** | Rap guanine nucleotide exchange factor (GEF) 5 | RAPGEF5 | 0.664 | 0.002 |
| **11755799_a_at** | tyrosine kinase with immunoglobulin-like and EGF-like domains 1 | TIE1 | 0.663 | 0.020 |
| **11736551_x_at** | v-rel reticuloendotheliosis viral oncogene homolog (avian) | REL | 0.662 | 0.007 |
| **11725198_at** | interleukin 1, alpha | IL1A | 0.661 | 0.014 |
| **11747602_a_at** | mitogen-activated protein kinase kinase kinase 8 | MAP3K8 | 0.661 | 0.023 |
| **11738209_a_at** | G protein-coupled receptor 17 | GPR17 | 0.660 | 0.005 |
| **11720062_s_at** | immediate early response 3 | IER3 | 0.655 | 0.000 |
| **11727504_at** | SRY (sex determining region Y)-box 7 | SOX7 | 0.654 | 0.005 |
| **11729058_s_at** | nuclear receptor subfamily 4, group A, member 3 | NR4A3 | 0.649 | 0.008 |
| **11717682_a_at** | guanine nucleotide binding protein (G protein), alpha 13 | GNA13 | 0.637 | 0.048 |
| **11723556_x_at** | DDB1 and CUL4 associated factor 16 | DCAF16 | 0.635 | 0.029 |
| **11726492_a_at** | ATP-binding cassette, sub-family G (WHITE), member 1 | ABCG1 | 0.628 | 0.005 |
| **11717823_s_at** | tumor necrosis factor, alpha-induced protein 2 | TNFAIP2 | 0.628 | 0.001 |
| **11746244_a_at** | interleukin 17 receptor C | IL17RC | 0.625 | 0.047 |
| **11748100_a_at** | dihydropyrimidinase-like 2 | DPYSL2 | 0.622 | 0.036 |
| **11719673_a_at** | thrombomodulin | THBD | 0.619 | 0.008 |
| **11724540_a_at** | von Willebrand factor | VWF | 0.616 | 0.008 |
| **11745516_a_at** | syntaxin 16 | STX16 | 0.611 | 0.027 |
| **11743000_at** | CD83 molecule | CD83 | 0.605 | 0.004 |
| **11756604_a_at** | protocadherin beta 2 | PCDHB2 | 0.600 | 0.004 |
| **11732999_a_at** | intercellular adhesion molecule 1 | ICAM1 | 0.600 | 0.005 |
| **11742103_a_at** | transducin-like enhancer of split 2 (E(sp1) homolog, Drosophila) | TLE2 | 0.599 | 0.007 |
| **11746433_a_at** | claudin 1 | CLDN1 | 0.597 | 0.006 |
| **11747499_a_at** | intercellular adhesion molecule 1 | ICAM1 | 0.597 | 0.004 |
| **11740245_a_at** | parathyroid hormone-like hormone | PTHLH | 0.597 | 0.016 |
| **11758013_s_at** | chromosome 8 open reading frame 4 | C8orf4 | 0.597 | 0.026 |
| **11761476_at** | v-ets erythroblastosis virus E26 oncogene homolog (avian) | ERG | 0.597 | 0.007 |
| **11724306_at** | ELL associated factor 1 | EAF1 | 0.595 | 0.001 |
| **11726611_x_at** | v-maf musculoaponeurotic fibrosarcoma oncogene homolog F (avian) | MAFF | 0.588 | 0.001 |
| **11717854_a_at** | zinc finger and SCAN domain containing 18 | ZSCAN18 | 0.585 | 0.003 |
| **11724346_a_at** | interferon induced with helicase C domain 1 | IFIH1 | 0.585 | 0.031 |
| **11733000_at** | intercellular adhesion molecule 1 | ICAM1 | 0.584 | 0.007 |
| **11733007_at** | angiomotin like 2 | AMOTL2 | 0.580 | 0.000 |
| **11749726_x_at** | nuclear receptor subfamily 4, group A, member 1 | NR4A1 | 0.578 | 0.003 |
| **11724665_at** | apelin | APLN | 0.577 | 0.003 |
| **11743730_at** | tumor necrosis factor (ligand) superfamily, member 10 | TNFSF10 | 0.575 | 0.007 |
| **11742063_a_at** | butyrophilin, subfamily 3, member A1 | BTN3A1 | 0.573 | 0.001 |
| **11715164_s_at** | immunoglobulin lambda-like polypeptide 3, pseudogene | IGLL3P | 0.573 | 0.006 |
| **11716396_a_at** | chromosome 10 open reading frame 26 | C10orf26 | 0.570 | 0.001 |
| **11724475_a_at** | microtubule associated tumor suppressor 1 | MTUS1 | 0.569 | 0.008 |
| **11716974_a_at** | pyruvate dehydrogenase kinase, isozyme 4 | PDK4 | 0.568 | 0.001 |
| **11753790_x_at** | ST3 beta-galactoside alpha-2,3-sialyltransferase 3 | ST3GAL3 | 0.568 | 0.008 |
| **11734026_at** | zinc finger protein 440 | ZNF440 | 0.568 | 0.036 |
| **11715267_s_at** | epiplakin 1 | EPPK1 | 0.567 | 0.011 |
| **11760894_s_at** | serine/arginine-rich splicing factor 5 | SRSF5 | 0.567 | 0.017 |
| **11717065_a_at** | gremlin 1 | GREM1 | 0.566 | 0.035 |
| **11759618_a_at** | solute carrier family 25, member 37 | SLC25A37 | 0.564 | 0.000 |
| **11727927_a_at** | forkhead box O1 | FOXO1 | 0.564 | 0.004 |
| **11748771_a_at** | YTH domain family, member 3 | YTHDF3 | 0.563 | 0.002 |
| **11723943_x_at** | pituitary tumor-transforming 1 interacting protein | PTTG1IP | 0.563 | 0.040 |
| **11733006_at** | angiomotin like 2 | AMOTL2 | 0.562 | 0.001 |
| **11730320_a_at** | neural precursor cell expressed, developmentally down-regulated 9 | NEDD9 | 0.562 | 0.002 |
| **11725057_s_at** | neural precursor cell expressed, developmentally down-regulated 9 | NEDD9 | 0.561 | 0.023 |
| **11716771_s_at** | salt-inducible kinase 1 | SIK1 | 0.561 | 0.001 |
| **11723370_s_at** | SH3 and multiple ankyrin repeat domains 3 | SHANK3 | 0.560 | 0.001 |
| **11723942_s_at** | pituitary tumor-transforming 1 interacting protein | PTTG1IP | 0.556 | 0.024 |
| **11750334_a_at** | v-rel reticuloendotheliosis viral oncogene homolog (avian) | REL | 0.548 | 0.002 |
| **11722162_a_at** | polo-like kinase 2 | PLK2 | 0.548 | 0.010 |
| **11744292_at** | NGFI-A binding protein 2 (EGR1 binding protein 2) | NAB2 | 0.547 | 0.013 |
| **11746463_a_at** | interleukin 6 (interferon, beta 2) | IL6 | 0.545 | 0.019 |
| **11757513_at** | nuclear factor of kappa light polypeptide gene enhancer in B-cells inhibitor, zeta | NFKBIZ | 0.544 | 0.008 |
| **11725983_at** | basic helix-loop-helix family, member e40 | BHLHE40 | 0.544 | 0.005 |
| **11734741_x_at** | LIM domain kinase 2 | LIMK2 | 0.543 | 0.003 |
| **11735114_at** | CCR4-NOT transcription complex, subunit 4 | CNOT4 | 0.541 | 0.001 |
| **11722052_at** | plasmolipin | PLLP | 0.538 | 0.007 |
| **11725793_s_at** | prostaglandin E receptor 4 (subtype EP4) | PTGER4 | 0.537 | 0.008 |
| **11752993_a_at** | dual specificity phosphatase 1 | DUSP1 | 0.532 | 0.007 |
| **11750671_s_at** | multimerin 2 | MMRN2 | 0.527 | 0.025 |
| **11759730_a_at** | ovostatin 2 | OVOS2 | 0.527 | 0.009 |
| **11723215_s_at** | myocyte enhancer factor 2C | MEF2C | 0.526 | 0.031 |
| **11722752_a_at** | chromosome 14 open reading frame 43 | C14orf43 | 0.526 | 0.001 |
| **11751016_s_at** | SRY (sex determining region Y)-box 7 | SOX7 | 0.525 | 0.002 |
| **11756358_a_at** | polo-like kinase 3 | PLK3 | 0.525 | 0.009 |
| **11715412_a_at** | endothelial PAS domain protein 1 | EPAS1 | 0.525 | 0.035 |
| **11716327_x_at** | gap junction protein, alpha 1, 43kDa | GJA1 | 0.524 | 0.031 |
| **11717619_at** | EPH receptor A2 | EPHA2 | 0.522 | 0.002 |
| **11747952_x_at** | tumor necrosis factor (ligand) superfamily, member 10 | TNFSF10 | 0.522 | 0.021 |
| **11753359_x_at** | phosphatidylinositol glycan anchor biosynthesis, class A | PIGA | 0.522 | 0.021 |
| **11724038_a_at** | prostaglandin-endoperoxide synthase 2 (prostaglandin G/H synthase and cyclooxygenase) | PTGS2 | 0.520 | 0.048 |
| **11744660_s_at** | chemokine (C-C motif) ligand 4-like 1 /// chemokine (C-C motif) ligand 4-like 2 | CCL4L1 /// CCL4L2 | 0.519 | 0.029 |
| **11749348_a_at** | solute carrier family 25 (mitochondrial carrier, Aralar), member 12 | SLC25A12 | 0.519 | 0.019 |
| **11718319_at** | CD93 molecule | CD93 | 0.516 | 0.026 |
| **11747516_a_at** | exosome component 10 | EXOSC10 | 0.516 | 0.011 |
| **11733417_x_at** | P antigen family, member 5 (prostate associated) | PAGE5 | 0.515 | 0.007 |
| **11745039_a_at** | aarF domain containing kinase 2 | ADCK2 | 0.515 | 0.017 |
| **11722163_x_at** | polo-like kinase 2 | PLK2 | 0.514 | 0.017 |
| **11721034_at** | protein phosphatase 1, regulatory (inhibitor) subunit 3B | PPP1R3B | 0.512 | 0.001 |
| **11721573_a_at** | activin A receptor type II-like 1 | ACVRL1 | 0.512 | 0.007 |
| **11724948_at** | Cbp/p300-interacting transactivator, with Glu/Asp-rich carboxy-terminal domain, 4 | CITED4 | 0.511 | 0.001 |
| **11725515_a_at** | ATP-binding cassette, sub-family G (WHITE), member 1 | ABCG1 | 0.511 | 0.009 |
| **11723555_at** | DDB1 and CUL4 associated factor 16 | DCAF16 | 0.510 | 0.050 |
| **11720341_at** | NADH dehydrogenase (ubiquinone) 1 alpha subcomplex, 13 | NDUFA13 | 0.509 | 0.002 |
| **11733420_x_at** | zinc finger protein 563 | ZNF563 | 0.508 | 0.007 |
| **11731894_a_at** | potassium intermediate/small conductance calcium-activated channel, subfamily N, member 3 | KCNN3 | 0.506 | 0.014 |
| **11750037_a_at** | CHMP family, member 7 | CHMP7 | 0.505 | 0.037 |
| **11764030_x_at** | CCAAT/enhancer binding protein (C/EBP), delta | CEBPD | 0.505 | 0.039 |
| **11719634_a_at** | Kruppel-like factor 4 (gut) | KLF4 | 0.504 | 0.041 |
| **11763227_at** | chromosome 6 open reading frame 48 /// small nucleolar RNA, C/D box 48 | C6orf48 /// SNORD48 | 0.502 | 0.022 |
| **11716194_a_at** | SET domain containing 2 | SETD2 | -1.002 | 0.002 |
| **11739969_a_at** | mesoderm induction early response 1 homolog (Xenopus laevis) | MIER1 | -1.009 | 0.000 |
| **11724809_at** | Cas-Br-M (murine) ecotropic retroviral transforming sequence | CBL | -1.022 | 0.000 |
| **11716766_a_at** | IQ motif containing GTPase activating protein 1 | IQGAP1 | -1.024 | 0.005 |
| **11728041_at** | somatostatin receptor 1 | SSTR1 | -1.056 | 0.006 |
| **11755635_s_at** | cyclin-dependent kinase 11A /// cyclin-dependent kinase 11B /// cell division protein kinase 11B-like /// cell division protein kinase 11B-like | CDK11A /// CDK11B /// LOC100133692 /// LOC100294398 | -1.066 | 0.003 |
| **11745119_a_at** | dystonin | DST | -1.069 | 0.007 |
| **11731624_a_at** | chromosome 10 open reading frame 118 | C10orf118 | -1.069 | 0.002 |
| **11742931_at** | baculoviral IAP repeat-containing 6 | BIRC6 | -1.119 | 0.006 |
| **11749826_a_at** | deiodinase, iodothyronine, type II | DIO2 | -1.155 | 0.000 |
| **11723756_at** | zinc finger RNA binding protein | ZFR | -1.210 | 0.001 |
| **11743281_a_at** | WNK lysine deficient protein kinase 1 | WNK1 | -1.221 | 0.008 |
| **11716566_a_at** | HLA-B associated transcript 2-like 2 | BAT2L2 | -1.234 | 0.005 |
| **11727136_a_at** | pseudouridylate synthase 7 homolog (S. cerevisiae)-like | PUS7L | -1.244 | 0.001 |

**Supplementary table 2. Regulated genes of HMEC-1** cells after exposure to trophoblastic debris for 21 hours.

| **Probe Set ID** | **Gene Title** | **Gene Symbol** | **log2 fold change** | **p value** |
| --- | --- | --- | --- | --- |
| **11742994_x_at** | chorionic somatomammotropin hormone 1 (placental lactogen) | CSH1 | 2.782 | 0.000 |
| **11734709_x_at** | chorionic somatomammotropin hormone 1 (placental lactogen) /// chorionic somatomammotropin hormone 2 | CSH1 /// CSH2 | 2.696 | 0.002 |
| **11745920_x_at** | chorionic somatomammotropin hormone 1 (placental lactogen) /// chorionic somatomammotropin hormone 2 | CSH1 /// CSH2 | 2.459 | 0.001 |
| **11743032_at** | glycoprotein hormones, alpha polypeptide | CGA | 2.049 | 0.004 |
| **11716630_a_at** | angiopoietin-like 4 | ANGPTL4 | 1.636 | 0.000 |
| **11727790_x_at** | secreted phosphoprotein 1 | SPP1 | 1.445 | 0.001 |
| **11754604_x_at** | secreted phosphoprotein 1 | SPP1 | 1.341 | 0.001 |
| **11746506_a_at** | secreted phosphoprotein 1 | SPP1 | 1.182 | 0.005 |
| **11716974_a_at** | pyruvate dehydrogenase kinase, isozyme 4 | PDK4 | 1.151 | 0.000 |
| **11739787_a_at** | insulin-like growth factor 2 (somatomedin A) /// INS-IGF2 readthrough transcript | IGF2 /// INS-IGF2 | 1.104 | 0.001 |
| **11742406_x_at** | chorionic somatomammotropin hormone 1 (placental lactogen) | CSH1 | 0.983 | 0.000 |
| **11737944_x_at** | secreted phosphoprotein 1 | SPP1 | 0.941 | 0.004 |
| **11742745_a_at** | perilipin 2 | PLIN2 | 0.917 | 0.003 |
| **11742746_a_at** | perilipin 2 | PLIN2 | 0.834 | 0.002 |
| **11717386_s_at** | metallothionein 1G | MT1G | 0.805 | 0.013 |
| **11757022_x_at** | tissue factor pathway inhibitor 2 | TFPI2 | 0.798 | 0.004 |
| **11755928_s_at** | matrix metallopeptidase 1 (interstitial collagenase) | MMP1 | 0.781 | 0.020 |
| **11755730_x_at** | secreted phosphoprotein 1 | SPP1 | 0.777 | 0.017 |
| **11727655_s_at** | dystonin | DST | 0.774 | 0.021 |
| **11746856_a_at** | serpin peptidase inhibitor, clade E (nexin, plasminogen activator inhibitor type 1), member 1 | SERPINE1 | 0.767 | 0.021 |
| **11737373_at** | t-complex 11 (mouse)-like 2 | TCP11L2 | 0.749 | 0.001 |
| **11718347_a_at** | S100 calcium binding protein P | S100P | 0.744 | 0.007 |
| **11715636_a_at** | serpin peptidase inhibitor, clade E (nexin, plasminogen activator inhibitor type 1), member 1 | SERPINE1 | 0.720 | 0.005 |
| **11722850_a_at** | baculoviral IAP repeat-containing 3 | BIRC3 | 0.718 | 0.031 |
| **11762578_a_at** | MKL/myocardin-like 2 | MKL2 | 0.710 | 0.037 |
| **11737662_a_at** | tet oncogene family member 2 | TET2 | 0.710 | 0.002 |
| **11743515_s_at** | laminin, gamma 2 | LAMC2 | 0.704 | 0.022 |
| **11715893_s_at** | interferon, alpha-inducible protein 27 | IFI27 | 0.696 | 0.021 |
| **11737943_a_at** | secreted phosphoprotein 1 | SPP1 | 0.687 | 0.018 |
| **11742698_at** | secretogranin V (7B2 protein) | SCG5 | 0.687 | 0.040 |
| **11743304_a_at** | eyes absent homolog 3 (Drosophila) | EYA3 | 0.680 | 0.003 |
| **11752333_a_at** | integrin, alpha V (vitronectin receptor, alpha polypeptide, antigen CD51) | ITGAV | 0.679 | 0.015 |
| **11727031_a_at** | sequestosome 1 | SQSTM1 | 0.678 | 0.004 |
| **11756003_x_at** | insulin-like growth factor 2 (somatomedin A) /// INS-IGF2 readthrough transcript | IGF2 /// INS-IGF2 | 0.675 | 0.023 |
| **11747923_s_at** | laminin, gamma 2 | LAMC2 | 0.672 | 0.020 |
| **11732469_at** | interleukin 3 receptor, alpha (low affinity) | IL3RA | 0.657 | 0.000 |
| **11760705_a_at** | NADH dehydrogenase (ubiquinone) Fe-S protein 2, 49kDa (NADH-coenzyme Q reductase) | NDUFS2 | 0.647 | 0.035 |
| **11763226_x_at** | interleukin 8 | IL8 | 0.632 | 0.041 |
| **11745458_a_at** | eyes absent homolog 3 (Drosophila) | EYA3 | 0.627 | 0.017 |
| **11735924_s_at** | keratin associated protein 12-1 /// keratin associated protein 12-2 | KRTAP12-1 /// KRTAP12-2 | 0.626 | 0.009 |
| **11754026_a_at** | interleukin 8 | IL8 | 0.626 | 0.043 |
| **11742653_x_at** | chorionic somatomammotropin hormone 1 (placental lactogen) /// chorionic somatomammotropin hormone 2 | CSH1 /// CSH2 | 0.625 | 0.044 |
| **11759629_a_at** | T cell receptor alpha constant /// T cell receptor alpha joining 17 /// T cell receptor alpha variable 20 | TRAC /// TRAJ17 /// TRAV20 | 0.620 | 0.034 |
| **11719916_at** | interleukin 1, beta | IL1B | 0.616 | 0.019 |
| **11743514_a_at** | laminin, gamma 2 | LAMC2 | 0.607 | 0.013 |
| **11754524_x_at** | tissue factor pathway inhibitor 2 | TFPI2 | 0.603 | 0.024 |
| **11760491_at** | caspase 4, apoptosis-related cysteine peptidase | CASP4 | 0.601 | 0.009 |
| **11722851_at** | baculoviral IAP repeat-containing 3 | BIRC3 | 0.601 | 0.049 |
| **11719120_a_at** | kynureninase (L-kynurenine hydrolase) | KYNU | 0.599 | 0.008 |
| **11734297_s_at** | zinc finger, X-linked, duplicated A /// zinc finger, X-linked, duplicated B | ZXDA /// ZXDB | 0.599 | 0.001 |
| **11754375_s_at** | sorting nexin 10 | SNX10 | 0.596 | 0.009 |
| **11737410_at** | chromosome 4 open reading frame 36 | C4orf36 | 0.594 | 0.000 |
| **11759765_at** | phospholipid scramblase 1 | PLSCR1 | 0.594 | 0.006 |
| **11759644_at** | oncostatin M receptor | OSMR | 0.593 | 0.011 |
| **11739197_a_at** | polycomb group ring finger 3 | PCGF3 | 0.582 | 0.002 |
| **11729052_at** | PTPRF interacting protein, binding protein 1 (liprin beta 1) | PPFIBP1 | 0.579 | 0.017 |
| **11763019_x_at** | proline-rich nuclear receptor coactivator 1 | PNRC1 | 0.576 | 0.000 |
| **11762881_at** | acylglycerol kinase | AGK | 0.572 | 0.001 |
| **11760318_at** | cytochrome P450, family 4, subfamily V, polypeptide 2 | CYP4V2 | 0.566 | 0.000 |
| **11715711_a_at** | aldo-keto reductase family 1, member C3 (3-alpha hydroxysteroid dehydrogenase, type II) | AKR1C3 | 0.564 | 0.002 |
| **11755438_x_at** | pregnancy specific beta-1-glycoprotein 3 | PSG3 | 0.559 | 0.010 |
| **11749948_x_at** | hydroxysteroid (17-beta) dehydrogenase 1 | HSD17B1 | 0.556 | 0.001 |
| **11733175_a_at** | zinc finger protein 507 | ZNF507 | 0.550 | 0.003 |
| **11733745_a_at** | glucosaminyl (N-acetyl) transferase 2, I-branching enzyme (I blood group) | GCNT2 | 0.548 | 0.002 |
| **11736135_at** | 2'-5'-oligoadenylate synthetase 2, 69/71kDa | OAS2 | 0.547 | 0.022 |
| **11749956_a_at** | WD repeat domain 67 | WDR67 | 0.547 | 0.001 |
| **11749640_a_at** | tankyrase, TRF1-interacting ankyrin-related ADP-ribose polymerase | TNKS | 0.547 | 0.003 |
| **11725749_a_at** | galanin prepropeptide | GAL | 0.546 | 0.034 |
| **11758803_at** | single-stranded DNA binding protein 2 | SSBP2 | 0.545 | 0.006 |
| **11737117_at** | poly(A) binding protein, cytoplasmic 5 | PABPC5 | 0.542 | 0.008 |
| **11734165_x_at** | Rho guanine nucleotide exchange factor (GEF) 26 | ARHGEF26 | 0.540 | 0.000 |
| **11753664_x_at** | RAD52 motif 1 | RDM1 | 0.539 | 0.002 |
| **11764075_at** | --- | --- | 0.539 | 0.006 |
| **11741745_a_at** | v-abl Abelson murine leukemia viral oncogene homolog 2 | ABL2 | 0.532 | 0.024 |
| **11750818_a_at** | tetratricopeptide repeat domain 39B | TTC39B | 0.529 | 0.015 |
| **11724169_s_at** | zinc finger protein 322A /// zinc finger protein 322B | ZNF322A /// ZNF322B | 0.528 | 0.004 |
| **11740206_x_at** | G protein-coupled receptor 155 | GPR155 | 0.528 | 0.000 |
| **11735095_at** | leptin | LEP | 0.527 | 0.014 |
| **11724419_at** | zinc finger CCCH-type containing 6 | ZC3H6 | 0.526 | 0.008 |
| **AFFX-r2-TagQ-3_at** | --- | --- | 0.525 | 0.030 |
| **11735302_a_at** | G protein-coupled receptor 123 | GPR123 | 0.521 | 0.001 |
| **11715673_x_at** | jun B proto-oncogene | JUNB | 0.516 | 0.003 |
| **11749589_x_at** | cathepsin S | CTSS | 0.516 | 0.003 |
| **11752091_a_at** | thyroid adenoma associated | THADA | 0.515 | 0.006 |
| **11749913_a_at** | heat shock protein 70kDa family, member 13 | HSPA13 | 0.514 | 0.016 |
| **11753515_a_at** | interleukin 32 | IL32 | 0.513 | 0.010 |
| **11761888_x_at** | pregnancy specific beta-1-glycoprotein 9 | PSG9 | 0.513 | 0.026 |
| **11738045_a_at** | lecithin retinol acyltransferase (phosphatidylcholine--retinol O-acyltransferase) | LRAT | 0.509 | 0.003 |
| **11761757_at** | FXYD domain containing ion transport regulator 7 | FXYD7 | 0.506 | 0.004 |
| **11761139_at** | aspartate beta-hydroxylase | ASPH | 0.505 | 0.002 |
| **11729540_at** | non-SMC condensin II complex, subunit H2 | NCAPH2 | 0.503 | 0.002 |
| **11744006_a_at** | vinculin | VCL | 0.500 | 0.003 |
| **11743519_at** | death inducer-obliterator 1 | DIDO1 | -1.324 | 0.000 |

**Supplementary table 3. Identification of differentially expressed proteins in HMEC-1 cells after exposure trophoblastic debris for** 24 hours.

| **Protein GI accessions** | **Protein Name** | **Gene name** | **iTRAQ ratios 1 treated vs. control** | **iTRAQ ratios2 treated vs. control** | **iTRAQ ratios 3 treated vs. control** |
| --- | --- | --- | --- | --- | --- |
| 66346730;  10334859 | Creatine kinase U-type, mitochondrial precursor | CKMT | 2.03 | 10.42 | 4.29 |
| 119392083 | Corticosteroid 11-beta-dehydrogenase isozyme 2 | HSD11B2 | Not detected | 5.69 | 3.83 |
| 4504919 | Keratin, type II cytoskeletal 8 | K2C8 | 2.07 | 6 | 2.42 |
| 5174663 | Protein S100-P | S100P | Not detected | 2.72 | 4.02 |
| 10334861 | Chorionic somatomammotropin hormone | CSH1/CSH2 | 2.59 | 4.74 | 2.41 |
| 401871059 | Tissue factor pathway inhibitor 2 isoform 2 precursor | TFPI2 | Not detected | 2.89 | 3.09 |
| 28302131 | Hemoglobin, gamma A | HBG1 | Not detected | 2.25 | 2.67 |
| 296317348 | Pregnancy-specific beta-1-glycoprotein 1 | PSG1 | 1.75 | 3.05 | Not detected |
| 47419900 | Pregnancy-specific beta-1-glycoprotein 4 | PSG4 | Not detected | 2.67 | 2.03 |
| 6715607 | Hemoglobin subunit gamma-2 | HBG2 | 1.3 | 2.81 | 2.75 |
| 24234699 | Keratin, type I cytoskeletal 19 | KRT19 | 1.3 | 2.97 | 2.17 |
| 109240546 | Pregnancy-specific beta-1-glycoprotein 3 | PSG3 | 1.88 | 2.1 | Not detected |
| 53831991 | Steryl-sulfatase | STS | Not detected | 1.9 | 2.04 |
| 4557871 | Serotransferrin | TF | 1.85 | 3.03 | 1.02 |
| 49574502 | NADH-cytochrome b5 reductase 1 | CYB5R1 | Not detected | 1.27 | 2.36 |
| 4502105 | Annexin A4 | ANXA4 | 1.44 | 2.22 | 1.72 |
| 4504349 | Hemoglobin subunit beta | HBB | 1.2 | 2.16 | 1.82 |
| 4505595 | Plasminogen activator inhibitor 2 | PAI2 | 1.62 | 1.71 | 1.66 |
| 4506773 | Protein S100-A9 | S100A9 | Not detected | 1.57 | 1.59 |
| 189458817 | Transferrin receptor protein 1 | TFRC | 1.51 | 1.59 | 1.55 |
| 4557888 | Keratin, type I cytoskeletal 18 | KRT18 | 1.09 | 1.65 | 1.81 |
| 4557735 | Amine oxidase [flavin-containing] A | MAOA | Not detected | 1.2 | 1.83 |
| 167466198 | Intercellular adhesion molecule 1 | ICAM1 | 1.13 | 1.25 | 2.09 |
| 10835159 | Plasminogen activator inhibitor 1 | PAI1 | 1.26 | 1.48 | 1.65 |
| 71773329 | Annexin A6 | ANXA6 | 1.4 | 1.47 | 1.43 |
| 156631005 | 26S proteasome non- ATPase regulatory subunit 8 | PSMD8 | 1.47 | 1.49 | 1.32 |
| 19913432 | V-type proton ATPase subunit d 1 | ATP6V0D1 | 1.38 | 1.32 | 1.51 |
| 166795299 | Solute carrier family 2, facilitated glucose transporter member 1 | SLC2A1 | 1.49 | 1.21 | 1.46 |
| 47078292 | Integrin beta-3 | ITGB3 | Not detected | 1.34 | 1.43 |
| 88853069 | Somatomedin-B | [SBSPON](http://www.genecards.org/cgi-bin/carddisp.pl?gene=SBSPON&search=deb77162cf51acc7799779c471f78f04) | 1.54 | 1.22 | Not detected |
| 4504345 | Hemoglobin subunit alpha | HBA | 1.02 | 1.22 | 1.82 |
| 117320527 | Nuclear factor NF-kappa-B p100 subunit | NFKB2 | Not detected | 1.24 | 1.45 |
| 70995211 | delta(3,5)-Delta(2,4)-dienoyl-CoA isomerase, mitochondrial precursor | ECH1 | 1.29 | 1.5 | 1.21 |
| 89264696 | Angiopoietin-related protein 4 | [ANGPTL4](http://www.genecards.org/cgi-bin/carddisp.pl?gene=ANGPTL4&search=4d4bddbd64fe4186fbee072ce63c5bbf) | 1.36 | 1.3 | Not detected |
| 19913418 | V-type proton ATPase 116 kDa subunit a isoform 1 | VPP1 | Not detected | 1.34 | 1.31 |
| 61742775 | Leucyl-cystinylaminopeptida se | [LNPEP](http://www.genecards.org/cgi-bin/carddisp.pl?gene=LNPEP&search=f48789c368058cc1a4a9fb61784113fb) | 1.44 | 1.17 | 1.35 |
| 31317224 | EGF-like repeat and discoidin I-like domain-containing protein 3 | EDIL3 | Not detected | 1.28 | 1.34 |
| 34577059 | Perilipin-2 | [PLIN2](http://www.genecards.org/cgi-bin/carddisp.pl?gene=PLIN2&search=3b5993a005c06e1c9d9190470368bec0) | 1.14 | 1.21 | 1.57 |
| 112380628 | Lysosome-associated membrane glycoprotein 1 | LAMP1 | 1.25 | 1.36 | Not detected |
| 195976754 | Dysferlin | DYSF | 1.13 | 1.29 | 1.43 |
| 54873613 | Agrin | AGRIN | 1.12 | 1.39 | 1.33 |
| 5902090 | Solute carrier family 2, facilitated glucose transporter member 3 | [SLC2A3](http://www.genecards.org/cgi-bin/carddisp.pl?gene=SLC2A3&search=2546c6781ddd55df1dcdfe5842175605) | Not detected | 1.29 | 1.24 |
| 127139033 | NADPH-cytochrome P450 reductase | CYP | 1.39 | 1.06 | 1.34 |
| 223468595 | integrin alpha-V isoform 2 precursor | ITGAV | 1.38 | 1.1 | 1.26 |
| 50980301 | Myosin phosphatase Rho-interacting protein | [MPRIP](http://www.genecards.org/cgi-bin/carddisp.pl?gene=MPRIP&search=7cf3387eb26578829e66f978dcee3f1c) | 1.03 | 1.39 | 1.27 |
| 39777597 | Protein-glutamine gamma- glutamyltransferase 2 | TGM2 | 1.41 | 0.98 | 1.3 |
| 299523086 | Mesencephalic astrocyte-derived neurotrophicfactor | [MANF](http://www.genecards.org/cgi-bin/carddisp.pl?gene=MANF&search=4bad8018fcfdbaaa564009f93d6b46a6) | 1.29 | 1.22 | 1.13 |
| 37594464 | ADP-sugar pyrophosphatase | NUDT5 | 1.22 | 1.09 | 1.24 |
| 4826852 | Acyl carrier protein, mitochondrial | NDUFAB1 | 1.22 | 1.01 | 1.31 |
| 154354966 | Mitochondrial inner membrane protein | IMMT | 0.8 | 0.72 | 1.28 |
| 47132595 | Phosphate carrier protein, mitochondrial | SLC25A3 | 0.76 | 0.72 | 1.14 |
| 42476281 | Voltage-dependent anion-selective channel protein 2 | VDAC2 | 0.79 | 0.69 | 1.12 |
| 4506617 | 60S ribosomal protein L17 | RPL17 | 0.76 | 0.71 | 0.93 |
| 4506685 | 40S ribosomal protein S13 | RPS13 | 0.77 | 0.73 | 0.86 |
| 156523970 | Alpha-2-HS- glycoprotein | FETUA | 0.72 | 1.25 | 0.38 |
| 195972866 | Keratin, type I cytoskeletal 10 | KRT10 | 0.54 | 1.24 | 0.45 |
| 119395750 | Keratin, type II cytoskeletal 1 | KRT1 | 0.56 | 1.15 | 0.5 |
| 49355721 | Protein FAM162A | FAM162A | 0.76 | 0.7 | Not detected |
| 47458041 | Calcium-binding mitochondrial carrier protein SCaMC-1 | SCaMC-1 | 0.68 | 0.54 | 0.92 |

**Supplementary table 4. Primer sequences for q**RT-PCR

| CSF2 | Forward | 5’-CTCAGAAATGTTTGACCTCCAG-3’ |
| --- | --- | --- |
|  | Reverse | 5’-TCAAAGGTGATAATCTGGGTTG-3’ |
| BIRC3 | Forward | 5’-CTGGAAAAGAGGAGACAGTCCT-3’ |
|  | Reverse | 5’-ACTGTTTTCTGTACCCGGAAGT-3’ |
| IL-8 | Forward | 5’-ACTTTCAGAGACAGCAGAGCAC-3’ |
|  | Reverse | 5’-CCAGCTTGGAAGTCATGTTTAC-3’ |
| ITGAV | Forward | 5’-CTTTCTTCCGATTCCAAACT-3’ |
|  | Reverse | 5’-CTTGCTGAATGAACTTGGAC-3’ |
| IFI27 | Forward | 5’-CTCTCTAGGCCACGGAATTAAC-3’ |
|  | Reverse | 5’-CACAACTCCTCCAATCACAACT-3’ |
| MMP1 | Forward | 5’-TTGATGAAGATGAAAGGTGGAC-3’ |
|  | Reverse | 5’-CACCACTGAAGGTGTAGCTAGG-3’ |
| CSH1 | Forward | 5’-TCCCAAAGGACCAGAAGTAT-3’ |
|  | Reverse | 5’-GGAGCAGCTCTAGATTGGAT-3’ |
| UBC | Forward | 5’-GGGCACTGGTTTTCTTTCCA-3’ |
|  | Reverse | 5’-AGAATCGCCGACAAGGGACTA-3’ |
| RPLP0 | Forward | 5’-ATGGGCAAGAACACCATGATG -3’ |
|  | Reverse | 5’-CCTCCTTGGTGAACACAAAGC -3’ |
| ACTB | Forward | 5’-GCGGACTATGACTTAGTTGCGTTA-3’ |
|  | Reverse | 5’-CATCTTGTTTTCTGCGCAAGTT-3’ |
| PPIA | Forward | 5’-GGGTTCCTGCTTTCACAGAATT-3’ |
|  | Reverse | 5’-GGACCCGTATGCTTTACCATGA-3’ |
